# The evolutionary rate of antibacterial drug targets

**DOI:** 10.1186/1471-2105-14-36

**Published:** 2013-02-01

**Authors:** Arkadiusz Gladki, Szymon Kaczanowski, Pawel Szczesny, Piotr Zielenkiewicz

**Affiliations:** 1 Institute of Biochemistry and Biophysics, Polish Academy of Sciences, Pawinskiego 5A, Warsaw, Poland; 2 Department of Plant Molecular Biology, Institute of Experimental Plant Biology and Biotechnology, University of Warsaw, Warsaw, Miecznikowa 1

## Abstract

**Background:**

One of the major issues in the fight against infectious diseases is the notable increase in multiple drug resistance in pathogenic species. For that reason, newly acquired high-throughput data on virulent microbial agents attract the attention of many researchers seeking potential new drug targets. Many approaches have been used to evaluate proteins from infectious pathogens, including, but not limited to, similarity analysis, reverse docking, statistical 3D structure analysis, machine learning, topological properties of interaction networks or a combination of the aforementioned methods. From a biological perspective, most essential proteins (knockout lethal for bacteria) or highly conserved proteins (broad spectrum activity) are potential drug targets. Ribosomal proteins comprise such an example. Many of them are well-known drug targets in bacteria. It is intuitive that we should learn from nature how to design good drugs. Firstly, known antibiotics are mainly originating from natural products of microorganisms targeting other microorganisms. Secondly, paleontological data suggests that antibiotics have been used by microorganisms for million years. Thus, we have hypothesized that good drug targets are evolutionary constrained and are subject of evolutionary selection. This means that mutations in such proteins are deleterious and removed by selection, which makes them less susceptible to random development of resistance. Analysis of the speed of evolution seems to be good approach to test this hypothesis.

**Results:**

In this study we show that pN/pS ratio of genes coding for known drug targets is significantly lower than the genome average and also lower than that for essential genes identified by experimental methods. Similar results are observed in the case of dN/dS analysis. Both analyzes suggest that drug targets tend to evolve slowly and that the rate of evolution is a better predictor of drugability than essentiality.

**Conclusions:**

Evolutionary rate can be used to score and find potential drug targets. The results presented here may become a useful addition to a repertoire of drug target prediction methods. As a proof of concept, we analyzed GO enrichment among the slowest evolving genes. These may become the starting point in the search for antibiotics with a novel mechanism.

## Background

Endless modifications of existing antibiotics might lead to the appearance of cross-resistance; therefore there is a need for parallel efforts of developing new types of antimicrobials. The preceding step is often finding a new drug target for these drugs. However, given the wealth of information provided by genome sequencing, mass spectrometry and microarray experiments, the selection of a potential drug target for pathogenic species and their relatives is not a trivial task. The repertoire of approaches starts with simple similarity searches, during which sequences of bacterial proteins are compared to known drug targets and human proteins [1]. Other methods are more extensive and implement analyses of metabolic and interaction networks [2] - approaches that, to our knowledge, were first tested with human protein drug targets [3]. There have been attempts to use machine learning methods to mine the substantial amount of data that can be found and derived for bacterial proteins. These approaches may focus on proteins as targets, providing lists of features (sequence length, mostly beta or alpha secondary structure, cytoplasmic/membrane bound, enzyme/non-enzyme, etc.) for a typical drug target [4]. Alternatively, they can focus on protein-protein interactions [5]. Finally, given a small molecule, one can dock it to the set of protein structures described in [6], which aids in the identification of new, potential drug targets for known compounds.

In general, all essential proteins of a given organism constitute potential drug targets [7,8]. The most prominent, essential proteins, such as the ribosomal proteins, are already approved drug targets in bacteria [9]. Their importance for the cell results in their evolutionary conservation [10]. For over 30 years, it has been commonly thought that essential genes are likely subject to stronger negative (purifying) selection (with a less frequent occurrence of mildly deleterious substitutions) [11] compared to nonessential genes. However, the prokaryotic and eukaryotic kingdoms seem to differ in this respect. No statistical difference in the relative rate of evolution between essential and nonessential genes was evident for the mouse, if immune genes were excluded [12]. Analysis of the yeast genome sheds light on eukaryotic species, by explaining why it was difficult to see a statistically significant difference in that case [13] (they observed a significantly higher rate of evolution of nonessential proteins when they compared essential proteins with the “most dispensable” half of nonessential proteins). In bacteria, stronger negative selection on essential genes was shown by Jordan [14] in the case of *Escherichia coli K12*. By applying an orthology-based essentiality transfer from *Escherichia coli*, the authors also predicted that this should be true for pathogenic species in the *Neisseria* and *Helicobacter genera*. This finding has been used for prediction of essential genes on its own or in conjunction with other methods.

No doubt we can learn a lot about choosing good drug targets from nature. Antibiotics are mainly originating from natural fungal and bacterial products [15]. Microorganisms have been using them for millions of years to combat (successfully) competing organisms. This impressive finding has been confirmed recently using paleontological data [16]. From the evolutionary point of view it may suggest that good drug targets are evolutionary constrained and are subject to purifying selection, which makes them less susceptible to random development of resistance. The efforts to validate this hypothesis and to find its application in drug design workflows comprise the aim of this study.

Analysis of evolutionary rates to identify putative drug targets has been already suggested by Searls [17], but no comprehensive study has been published so far. Moreover, two methods were proposed to identify evolutionary constrained residues in drug targets. Durand and co-workers [18] assessed purifying selection on individual sites in *Plasmodium falciparum* drug targets using the dN/dS ratio. The method is called “evolutionary patterning” (EP). A second method called “evolutionary tracing” (ET) [19], was proposed by Lichtarge in 1996. The Lichtarge method is based solely on evolutionary conservation. In both methods the key assumption is that a good drug should bind to the slowly evolving protein pocket. This expectation is based on intuition that at such sites development of drug resistance will be less probable. Both methods do not provide the overall picture of the evolutionary rates of genes of pathogenic species as they focus on individual sites, not the whole genes.

Generally speaking the key question we wanted to answer in this study was whether proteins which are targeted by antibiotics tend to evolve slowly. For this purpose we analyzed relative rate of evolution of genes from seven bacterial pathogens and from *E. coli*. We used polymorphism analysis, i.e. pN/pS ratio (which represents an appropriate measure of purifying selection in the case of comparison of inter-species diversity) and reproduced the analysis with dN/dS ratio (which is better for comparing sequences derived from different species) [20]. In both cases the rate of evolution of known drug targets, was not only significantly lower than the genome average but was also significantly lower than that for the essential genes, suggesting a higher selective force acting on a wide spectrum of drug targets. This finding suggests that calculation of evolutionary rate can aid in scoring during the process of drug target selection and can provide additional insights into whether a particular protein might or might not be an attractive drug target. As such, it complements EP/ET approaches.

## Methods

### Data preparation

The input set for our analysis consisted of bacterial genomes for which experimental data for the identification of essential genes existed (Table 1). Data on essential genes were obtained from the DEG database [21]. We obtained alignments of clusters of coding sequences (CDS) from whole-genome alignments of the reference genome (strain with experimental list of essential genes) and genomes of other strains from the ATGC (Alignable Tight Genomic Clusters) database [22].

**Table 1:** Summary of the data used at pN/pS analysis

**Reference genome (strain with experimental data on essential genes; NCBI Taxonomy ID in brackets)**	**Other strains with complete pairwise alignments with reference genome (NCBI Taxonomy IDs)**	The number of all genes^*^	The number of essential genes^*^	The number of drug targets^*^
*Escherichia coli K12 (*83333)	155864, 199310, 316407, 331111, 331112, 362663, 364106, 386585, 405955	4294	771	41
		(3104)	(616)	(31)
*Francisella novicida U112* (401614)	119857, 177416, 393011, 393115, 418136, 458234	1719	391	34
		(1065)	(320)	(32)
*Haemophilus influenzae Rd KW20* (71421)	262727, 262728, 281310, 374927, 374928, 374930, 374931, 374932, 374933, 375063, 375177, 375432	1581	477	39
		(1024)	(399)	(34)
*Helicobacter pylori 26695 (*85962)	357544, 85963	1576	336	30
		(992)	(292)	(28)
*Pseudomonas aeruginosa UCBPP-PA14 (*208963)	208963, 381754	5892	335	40
		(4530)	(305)	(36)
*Salmonella typhimurium LT2 (*99287)	209261, 220341, 295319, 321314	4425	481	41
		(3140)	(403)	(31)
*Staphylococcus aureus N315 (*93061)	158878, 158879, 196620, 273036, 282458, 282459, 359786, 359787, 367830, 418127, 426430, 93062	2892	351	35
		(1918)	(277)	(28)
*Streptococcus pneumoniae TIGR4 (*170187)	171101, 373153, 406556, 406557, 406558, 406559, 406560, 406561, 406562, 406563	1965	195	37
		(1532)	(175)	(33)

^*^All genes, essential genes and drug targets comprised the three groups of genes compared in our study. The final analysis was restricted to genes for which no recombination events were detected, numbers of which are presented in brackets.

For further comparison, we chose only reference genomes with at least two alignments with strains (subspecies) with complete genomes available in the ATGC database. The majority of cluster alignments we have obtained consisted of two sequences (one-to-one orthology assignments). However, in a small number of cases (~5%) we had more than one orthologous sequence representing a particular cluster in the compared genomes. Such duplications were resolved using reciprocal BLAST [23] on the corresponding protein sequences.

All genes were divided into three sets (see Table 1). The first group contained all genes from a particular organism, and the second group contained all of its essential genes (from the DEG database). The third group, referred to later as “potential wide-spectrum drug targets”, consisted of genes belonging to one of the orthology groups (as defined by KEGG KO [24]) covering bacterial drug targets with a known broad-spectrum activity acting as antagonist, inhibitor or in an adduct. In the case of duplications, (more than one gene in a single KO), the bidirectional best hit was selected using the KEGG SSDB database. The most comprehensive database containing FDA-approved existing drug targets is DrugBank [25]. We used provided data and then manually verified them. The verification included removal of beta-lactamases, which are drug targets and drug resistance enzymes at the same time which leads to a completely different evolutionary pattern than a typical drug target. However, we have included the enoyl-acyl carrier protein reductase (*fabI* gene), as this protein is a known drug target of the antibacterial agent Triclosan [26]. Data for Triclosan are not in DrugBank yet, however, this compound was used in an antibiotic profiling study in *Escherichia coli*[27], and it seemed reasonable to use it for further analysis. The final list of drug targets for each species is shown in additional table file (see Additional file 1).

All three groups were mutually exclusive. We removed the genes corresponding to known drug targets from the group of essential genes. Similarly, in the group of all genes, those known as lethal genes or known as drug targets were excluded. The rationale for such approach was to avoid biasing the p-value tests.

### Estimation of evolutionary rate

For each MSA of orthologous sequences, we evaluated polymorphism (the pN/pS ratio) using polyDnDs software [28]. We chose simple statistics based on a number of nonsynonymous and synonymous mutations (not taking into account number of possible places where mutations can occur).

### Assessment of pN/pS differences

For each species, we assessed the statistical difference of relative speed of evolution between the three aforementioned groups of genes (all, essential and potential wide-spectrum drug targets). We used the Mann–Whitney U test [29]. The p-values for a difference between sets were calculated using R implementation of the test (wilcox.test function; two sided).In our statistical approach we tested 24 hypotheses (three sets compared in one combination for eight species). We corrected our p-values using FDR approach (Benjamini-Yokutieli correction for multiple testing approach) [30].

### Gene ontology analysis

We used ontologies from Gene Ontology [31] (file gene_ontology_edit.obo; 10.07.2011), while annotations were obtained from EBI (Uniprot-GOA [32]). More than 60% of genes for all the species had at least one GO term assigned. For each species, 10% of the slowest evolving genes were selected as study set, while all genes in the species comprised the population set. Analysis was performed using command line version of Ontologizer [33].

#### *Plasmodium falciparum* pN/pS analysis

We estimated evolutionary rate of all *Plasmodium falciparum* genes and ranked them on this parameter. It enabled the assessment of evolutionary rate of the two genes used in the evolutionary patterning (EP) study, i.e. dihydrofolate synthase (DHFR-TS) and glycerol kinase (GK).

The pN/pS ratio was estimated using the approach proposed by Krzyczmonik et al. [34]. Thus we calculated pN/pS using nonsynonymous and synonymous SNPs from PlasmoDB [35]. We used SNP observable for the *Plasmodium falciparum 3d7* strain and other strains of this species. As it was shown by Krzyczmonik et al. [34] in many cases *P. falciparum* genes have only nonsynonymous genes and under such conditions it is impossible to calculate pN/pS ratio (as pS equals zero). We applied the correction suggested by those authors, i.e. in such cases pS were approximated by 1.

### Additional tests

In addition to the above analyses, we have conducted the tests using omega (dN/dS) instead of pN/pS. While such an approach is obviously biased, we were interested if the overall results would be different. Detailed methods, incorporating correction on recombinant genes, are provided in the supplementary materials.

## Results

### The evolutionary rate of potential wide-spectrum drug targets

**Figure 1:**
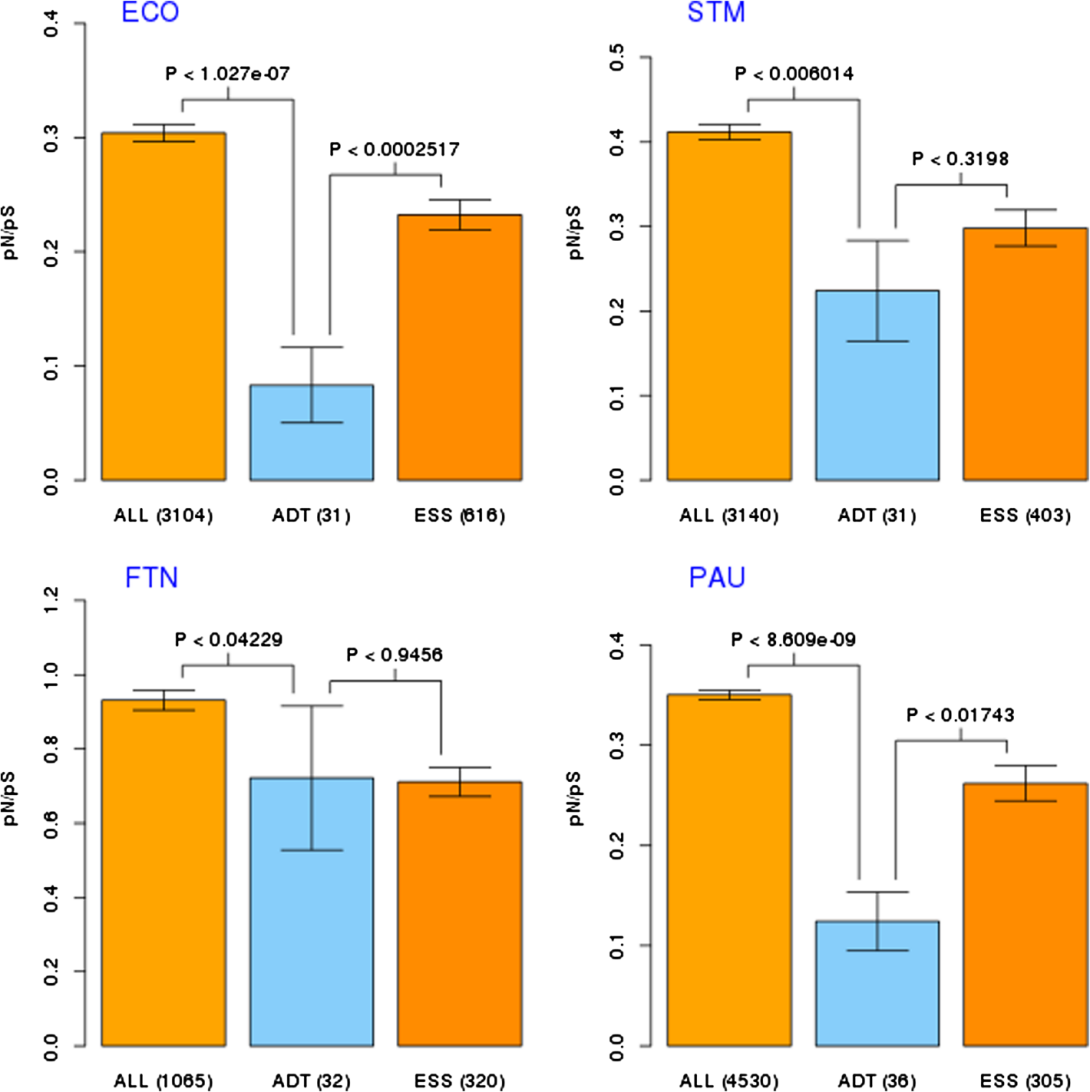
**Evolutionary rate differences of four Enterobacteriaceae species.** Evaluation of evolutionary rate differences between three sets of genes of interest: ALL -all genes, ESS - essential genes and ADT - approved drug targets). Evolutionary rate was estimated using (pN/pS ratio). In this case pN/pS values were compared using Mann–Whitney U test (wilcox.test in R language, two sided hypothesis tested). Box plots of means of pN/pS with 95% confidence intervals are presented (number of genes in given set are shown in brackets). Result for four species from *Enterobacteriaceae*. Abbreviations: ECO: *Escherichia coli*, STM – *Salmonella typhimurium*, PAU – *Pseudomonas aeruginosa*, FTN – *Francisella novicida.*

**Figure 2:**
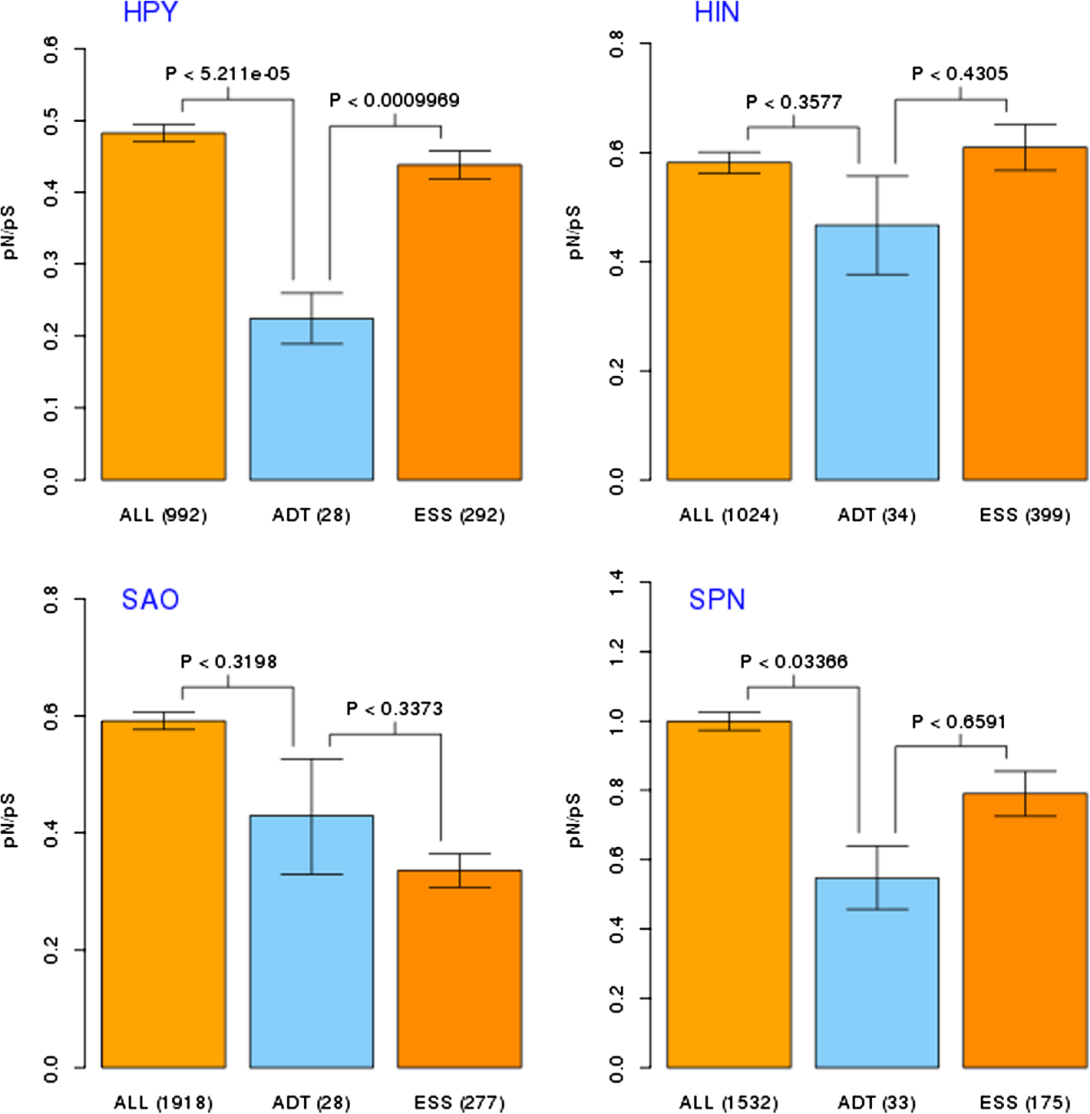
**Evolutionary rate differences of four non-Enterobacteriaceae species.** Evaluation of evolutionary rate differences between three sets of genes of interest: ALL -all genes, ESS - essential genes and ADT - approved drug targets). Evolutionary rate was estimated using (pN/pS ratio). In this case pN/pS values were compared using Mann–Whitney U test (wilcox.test in R language, two sided hypothesis tested). Box plots of means of pN/pS with 95% confidence intervals are presented (number of genes in given set are shown in brackets). Result for four species not from *Enterobacteriaceae* taxon. Abbreviations: HPY: *Helicobacter pylori*, HIN – *Haemophilus influenzae*, SAO – *Staphylococcus aureus*, SPN – *Streptococcus pneumoniae.*

We have analyzed genomes of seven pathogenic species. The results are summarized in Figure 1 and Figure 2 (see Additional file 2 for more statistical details). Potential drug targets had significantly lowered values of pN/pS compared to all genes from a given genome, as assessed by the average pN/pS ratio per orthologous group (although in the case of *S. aureus* and *H. influenzae* the differences were not statistically significant). Also, we observed lower pN/pS values for potential drug targets in comparison to essential genes (for all except *F. novicida* and *S. aureus*). In most genomes (all except *H. influenzae* and *H. pylori*), essential genes showed higher negative selection than the genome average, confirming the results of Jordan [14].

We also performed the same analysis on the genome of the non-pathogenic species, *Escherichia coli strain K12*. In this case, essential genes had lower pN/pS values than the genome average, and potential drug targets had lower pN/pS values than essential genes and the genome average. All those differences were highly significant (p-value < 0.01).

It should be noted, that the results of the analysis above are similar when using omega (dN/dS) instead of pN/pS. Details can be found in Supplementary Materials (see Additional file 3 and Additional file 4).

### Characterization of the orthology groups of drug targets

The assignment of known drug targets to KEGG KO orthology groups (see Methods) resulted in 44 representative groups (see Table 2). The three largest groups were various ribosomal proteins (~30%), proteins related to DNA processing and penicillin binding proteins. We ranked all of the orthology groups according to the average dN/dS of genes belonging to a given group across the eight genomes (seven pathogenic species and *Escherichia coli K12*) (see Additional file 1 for more details). Ribosomal and DNA processing enzymes ranked at the top and were the most attractive drug targets from an evolutionary perspective. The same could be said about penicillin binding protein (PBP) - 2 and PBP3 but not PBP4, PBP5/6 and PBP7. These results agree well with experimental data on these proteins [36]. PBP2 and PBP3 are bound by beta-lactam antibiotics with high affinity and are known to be the main/lethal target of these drugs. Affinity is lower in the cases of PBP4, PBP5/6 and PBP7. PBP4 to 7 contribute to penicillin resistance but are considered auxiliary drug targets.

**Table 2:** Ranking of known drug targets (from the evolutionary perspective)

**Drugs**	**KEGG KO**	**Mean pN/pS**	**SD**	**Gene name; description**
Cycloserine	ko:K01921	0.5813	0.136342	ddl; D-alanyl-alanine synthetase A
	ko:K01775	0.4017	0.260427	alr; alanine racemase
Triclosan	ko:K00208	0.2425	0.084719	fabI; enoyl-(acyl carrier protein) reductase
Trimethoprim	ko:K00287	0.6356	0.265319	folA; dihydrofolate reductase
Sulphonamides	ko:K00796	0.5708	0.213370	folP; dihydropteroate synthase
Fusidic acid	ko:K02355	0.1284	0.104805	fusA; elongation factor G
Mupirocin	ko:K01870	0.3031	0.139443	ileS; isoleucyl-tRNA synthetase
Quinolones	ko:K02469	0.3063	0.167897	gyrA; DNA gyrase subunit A
	ko:K02470	0.2195	0.192711	gyrB; DNA gyrase subunit B
	ko:K02621	0.3364	0.158633	parC; DNA topoisomerase IV subunit A
	ko:K02622	0.3016	0.231233	parE; DNA topoisomerase IV subunit B
Rifampin	ko:K03046	0.1263	0.047384	rpoC; DNA-directed RNA polymerase subunit beta'
	ko:K03043	0.1423	0.092346	rpoB; DNA-directed RNA polymerase subunit beta
	ko:K03040	0.4072	0.388638	rpoA; DNA-directed RNA polymerase subunit alpha
Macrolides	ko:K02926	0.2090	0.242629	rplD; 50S ribosomal protein L4
	ko:K02890	0.2212	0.408734	rplV; 50S ribosomal protein L22
	ko:K02864	0.4581	0.364803	rplJ; 50S ribosomal protein L10
	ko:K02911	0.5297	0.438268	rpmF; 50S ribosomal protein L32
Tetracyclines	ko:K02986	0.4107	0.343409	rpsD; 30S ribosomal protein S4
	ko:K02982	0.1927	0.187825	rpsC; 30S ribosomal protein S3
	ko:K02965	0.1139	0.171661	rpsS; 30S ribosomal protein S19
	ko:K02992	0.3173	0.327775	rpsG; 30S ribosomal protein S7
	ko:K02954	0.4495	0.467237	rpsN; 30S ribosomal protein S14
	ko:K02994	0.1578	0.203180	rpsH; 30S ribosomal protein S8
	ko:K02996	0.1692	0.216565	rpsI; 30S ribosomal protein S9
Glacycyline	ko:K02952	0.6584	0.422977	rpsM; 30S ribosomal protein S13
Retapamulin	ko:K02906	0.2112	0.307444	rplC; 50S ribosomal protein L3
Aminoglycosides	ko:K02946	0.1711	0.293128	rpsJ, nusE; 30S ribosomal protein S10
	ko:K02878	0.1300	0.151462	rplP; 50S ribosomal protein L16
	ko:K02950	0.4372	0.425007	rpsL; 30S ribosomal protein S12
beta-lactam antibiotics	ko:K07258	0.4060	0.280471	dacA; D-alanyl-D-alanine carboxypeptidase fraction A
	ko:K05515	0.2293	0.118958	mrdA; penicillin-binding protein 2
	ko:K03693	0.4693	0.000000	penicillin-binding protein 1B
	ko:K03587	0.2658	0.077185	ftsI; division specific transpeptidase (PBP3)
	ko:K00687	0.3279	0.000000	penicillin-binding protein 2B
	ko:K12553	0.4829	0.000000	penicillin-binding protein 3
	ko:K05365	0.3425	0.334610	mrcB, ponB; penicillin-binding protein 1B
	ko:K05366	0.3644	0.086939	mrcA; penicillin-binding protein 1A
	ko:K07262	0.3863	0.118514	pbpG; D-alanyl-D-alanine endopeptidase
	ko:K12556	0.3431	0.000000	penicillin-binding protein 2X
	ko:K12555	0.5859	0.000000	penicillin-binding protein 2A
	ko:K05367	0.7132	0.001414	pbpC; penicillin-binding protein 1C
	ko:K07259	0.3973	0.161482	dacB; D-alanyl-D-alanine carboxypeptidase/endopeptidase
Fosfomycin	ko:K00790	0.2551	0.183465	murA; UDP-N-acetylglucosamine 1-carboxyvinyltransferase

Drug targets are ordered by drug class. Mean pN/pS as well as standard deviation (SD) were calculated using data from eight species analyzed in the study.

Among protein groups with relatively high rate of evolution are alanine racemase and d-alanine ligase, which are targeted by sulphonamides [37]. Both of these proteins seem to be only moderately attractive drug targets from an evolutionary perspective. The D-alanine ligase gene has paralogs in *Escherichia coli* and *Salmonella typhimurium*, and such genes generally do not constitute good drug targets [38]. The same can be said about folate reductase and dihydropteroate synthase, two genes from the folate pathway targeted by two distinct classes of drugs. In the case of these genes, the fast appearance of resistance is commonly known [39]. Thus, drugs for these targets are often applied in combination. It is worth to note that all these proteins (alanine racemase, alanine ligase, folate reductase, dihydropteroate synthase) are drug targets of human designed antibiotics (i.e. synthetic as opposite to semisynthetic antibiotics being derivatives of bacterial natural products). Thus we see clearly how difficult it is to find a good novel drug target without referring to evolutionary history of pathogenic species. This is probably one of the key reasons why there has been no new class of antibiotic introduced into the market for the past twenty years [40].

### Functional classes among slowly evolving genes

Additionally, we analyzed slowly evolving genes by means of GO enrichment. Results partially overlap with common functional classes characterizing known drug targets (see Table 3). Statistically significant terms were “*rRNA binding*” and “*structural molecule activity*” corresponding to ribosomal proteins or “*nucleic acid binding*” corresponding to topoisomerases, RNA polymerases and gyrases. However we also identified some novel classes, usually not associated with known drug targets, such as succinate dehydrogenase or metal binding proteins. These may become interesting starting points in finding new drug targets with a unique mechanism of action.

**Table 3:** GO analysis for slowly evolving genes

**GO**	**P-value**	**Study-fraction**	**Population-fraction**	**Description**
GO:0005198	2.7e–37	0.00523	0.0359	structural molecule activity
GO:0005515	1.8e–12	0.0257	0.0685	protein binding
GO:0005488	2.9e–12	0.274	0.373	binding
GO:0019843	1.4e–06	0.0028	0.0194	rRNA binding
GO:0015453	0.00043	0.000997	0.00627	oxidoreduction-driven active transmembrane transporter activity
GO:0000104	0.0076	0.000436	0.00285	succinate dehydrogenase activity
GO:0016667	0.011	0.00567	0.012	oxidoreductase activity, acting on a sulfur group of donors
GO:0003735	0.014	0.00392	0.0308	structural constituent of ribosome
GO:0015078	0.027	0.00361	0.00856	hydrogen ion transmembrane transporter activity
GO:0046872	0.045	0.0756	0.105	metal ion binding

Enrichment of GO terms (molecular function domain): data for slowly evolving genes. Ten percent of genes (of eight studied species) with the lowest pN/pS rank comprised the study set. Genes with reliable pN/pS (of eight studied species) comprised the population set. Analysis was performed using Ontologizer (with Benjamini-Hochberg correction for multiple testing and Parent–child-Union Settings).

### Complementation of other evolutionary approaches for drug target discovery

In our opinion the approach presented here could be a good complementation to other drug target discovery methods based on evolutionary data: evolutionary tracing (ET) and evolutionary patterning (EP). As a proof of concept we analyzed the data of both approaches and put them into the context of our approach.

In the case of EP, Durand et al. analyzed the position specific evolutionary rate for two *Plasmodium falciparum* genes: known drug target, dihydrofolate reductase (DHFR-TS) and drug target candidate, glycerol kinase (GK). We estimated pN/pS genome wide, for almost all genes of *Plasmodium falciparum* (3d7 strain) and ranked genes using this parameter. Then we were able to evaluate those genes in the context of observed genome-wide distribution of pN/pS. We observed high purifying selection (slow evolutionary rate) for glycerol kinase (ranked in 32-nd percentile), which allows this gene to be considered as an attractive drug target from our perspective. We also observed rather weak purifying selection in the case of dihydrofolate reductase (ranked in 76-th percentile). It agrees well with the fact that antifolate resistance in the malaria parasite is well recognized [41]. Moreover, orthologous dihydrofolate reductases being known antibacterial drug targets are also under relatively weak selection pressure (as we pointed out earlier; see also Table 2).

In the case of evolutionary tracing (ET) Adikesavan et al. [42] presented in 2011 a first application of their approach to prokaryotes. They identified evolutionarily important surface amino acids involved in *Escherichia coli RecA* functions. *RecA* is already known as a drug target or co-drug target (in species for which gene knockout results in higher effectiveness of antibiotics). We compared evolutionary rate of the *recA* gene in the eight bacterial species analyzed in our study. In all cases (except for the *Streptococcus pneumoniae*) the *recA* gene was under strong evolutionary pressure (ranked in the lower quartile for these species and in the upper quartile in the case of *S. pneumoniae*) which makes it a good drug target from the perspective of our approach.

## Discussion

Antibiotics are mainly natural products used by micro-organisms against other micro-organisms. They seem to be relatively evolution proof, i.e. resistance is sufficiently rare and it is still beneficial for microorganism to use antibiotics against competing microorganisms.

We have shown that in most of the analyzed pathogenic genomes potential drug targets have statistically significant higher negative selection than essential genes or the genome average. Our explanation for this phenomenon is that such proteins are evolutionary constrained, i.e. they are overall highly sensitive to perturbations, which could correspond to relatively infrequent point mutations (including those leading to resistance).

One may ask the question whether our observation is not the result of the fact that bacteria have already been subject to considerable “drug” pressure - either by medical usage of compounds [43] or by more ancient and long term exposures to the natural products on which the antibiotics are based [44]. The observed purifying selection may, in this case, be the result of selective sweeps brought about by the drugs. If it would be the case more sensitive variants would be removed due to selection caused by the drug. The dN/dS analysis suggests that this possibility should be excluded. This test compares relative rate of evolution observed in the comparison of two closely related species. In contrast to the pN/pS test which takes in account existing allelic diversity, dN/dS takes into account only fixed mutations (with frequency = 1 in the population). Therefore, it is much more robust to the observed selective sweeps caused by antibiotics.

In some cases in our study we observed exceptions to the described general pattern. For example for *Staphylococcus aureus* and *Haemophilus influenzae* we did not observe a statistical difference in average pN/pS values between essential genes and potential drug targets (although drug targets evolve much slower than other genes). It is likely that for many drug targets, directed positive selection has led to intrinsic resistance; many *Staphylococcus aureus* strains are known to be resistant to vancomycin (VRSA) as well as methicillin (MRSA). Similarly, many *Haemophilus influenzae* strains have intrinsic resistance to beta-lactam drugs. It makes the observable differences in evolutionary rate between drug targets and other analyzed groups (essential genes and all genes) being lower than they in fact are.

Drug targets also have a higher negative selection when assessed by dN/dS ratio (omega). We consider these results supplementary as omega analysis has certain drawbacks (e.g. sensitiveness to recombination, limited range of dS for which dN/dS ratio is considered to be reliably estimated or non-linear dependency on time). Nevertheless, dN/dS analysis confirms the results obtained by the pN/pS approach.

When thinking about developing drugs against a certain target, one must also consider issues such as resistance mechanism (efflux pumps, other resistance proteins), drug target accessibility or host-related factors. Considering whether a gene is essential (even in a broader context than is commonly considered [45]) or conserved is often not sufficient. The assessment of evolutionary rate (e.g. by pN/pS values) helps substantially in the evaluation of potential drug targets. The resulting targets have an evolutionary history suggesting that they are less likely to randomly develop resistance via point mutations. And while it seems that the only cases one could find are the “obvious” ones, we show that this approach identified metal ion binding genes and succinate dehydrogenases - neither of which corresponds to well-studied wide-spectrum drug targets. Our results show that pN/pS analyses are an attractive addition to drug target prediction pipelines.

On the other hand one should be aware of the biases in our method. First, we used only whole genomes to limit the cases where low quality alignments will substantially affect pN/pS estimation. Because of that the eight chosen species are among the most common infective bacteria (they were among the first sequenced species). This, of course, is advantageous to address concerns like MRSA and VRSA and other antibiotic resistant pathogens, but then there is no evidence that the conclusions of this study apply to the less common human pathogens, to veterinary pathogens, or to other bacteria which could be antibiotic targeted. Second, we needed to limit the analysis to only eight species as the approach undertaken requires data on essential genes. This raises a concern whether Gram-negative species dominating in this study led to biased results. And finally, predicting evolutionary rate with pN/pS is limited to alignments of closely related sub-species. The last issue can be avoided by performing dN/dS analysis, but as we mentioned above dN/dS methodology has its own drawbacks.

## Conclusions

In this study we showed that good drug targets evolve slowly and that the rate of evolution is a better predictor of drugability than essentiality. This to some extent explains why known antibiotics (usually being of microbial origin) have been efficiently targeting other microorganisms for millions of years of evolution [16].

Our study also shows that evolutionary rate can be used to score and find potential drug targets. Generally our approach can be considered a useful complementation to EP (Evolutionary Patterning) and ET (Evolutionary Tracing) approaches. Both those methods can be useful in designing a drug that targets a specific site and has a known mechanism of operation. Our approach can be considered an attractive solution in the preceding step, i.e. finding the targets which could be analyzed in detail by ET or EP.

## Competing interests

The authors declare that they have no competing interests.

## Authors’ contributions

AG performed the analysis. AG, SK, PS and PZ participated in the design of the study. AG and PS drafted the manuscript. SK and PZ revised the manuscript. All authors read and approved the final manuscript.

## Supplementary Material

Additional file 1 **Essentiality and ranking of known drug targets.** The essentiality and ranking (from an evolutionary perspective) of known drug targets. Drug targets are ordered by drug class. Data for eight species of interest are presented in separate columns. Red: essential genes, blue: nonessential genes, grey: no data addressing essentiality, white: no ortholog with reliable omega. Click here for file

Additional file 2 **Statistical data - pN/pS analysis.** Mean pN/pS ratio for three sets of genes (ADT - drug targets, ESS - essential genes, ALL - other genes) presented. Data for eight species analyzed in the study. P-values where corrected for multiple testing with FDR (Benjamini-Yekutieli algorithm). Abbreviations: ciu - confidence interval upper limit; cil - confidence interval lower limit, eco – *Escherichia coli*, ftn – *Francisella novicida*, hin – *Haemophilus influenzae*, hpy – *Helicobacter pylori*, pau – *Pseudomonas aeruginosa*, sao – *Staphylococcus aureus,* spn – *Streptococcus pneumoniae*, stm – *Salmonella typhimurium.* Click here for file

Additional file 3 **Statistical data - dN/dS analysis.** Mean dN/dS ratio for three sets of genes (ADT - drug targets, ESS - essential genes, ALL - other genes) presented. Data for eight species analyzed in the study. P-values where corrected for multiple testing with FDR (Benjamini-Yekutieli algorithm). Abbreviations: ciu - confidence interval upper limit; cil - confidence interval lower limit, eco – *Escherichia coli*, ftn – *Francisella novicida*, hin – *Haemophilus influenzae*, hpy – *Helicobacter pylori*, pau – *Pseudomonas aeruginosa*, sao – *Staphylococcus aureus,* spn – *Streptococcus pneumoniae*, stm – *Salmonella typhimurium*. Click here for file

Additional file 4 **Dn/Ds analysis - materials, methods and results.** Analysis of evolutionary rate (dN/dS ratio) of known drug targets. Materials and methods as well as Results are presented in the file. Details about recombination (detection and removal procedures) can be also found. Click here for file
