# Supplementary material for: The evolutionary rate of antibacterial drug targets
Source: BMC Bioinformatics. 2013 Feb 1;14:36. doi: 10.1186/1471-2105-14-36 (PMC3598507; doi:10.1186/1471-2105-14-36)
Supplement: Additional file 4 — Dn/Ds analysis - materials, methods and results. Analysis of evolutionary rate (dN/dS ratio) of known drug targets. Materials and methods as well as Results are presented in the file. Details about recombination (detection and removal procedures) can be also found. [file 1471-2105-14-36-S4.doc]

**Selection of species/strains from ATGC cluster with acceptable evolutionary distance**

We restricted our workflow to bacterial species for which essential genes were identified experimentally (as we did for pN/pS analysis). These species were denoted as “reference” in Table 1 of the supplementary material.

From the ATGC database we have identified a cluster of closely related genomes for each “reference” genome. Then we conducted pairwise alignments of orthologous genes between the “reference” genome and each genome from the cluster.

For each pairwise alignment of orthologous sequences, we calculated omega values (the dN/dS ratio) using PAML (Phylogenetic Analysis by Maximum Likelihood) software with a run mode of -2. Codon frequencies were calculated from the average nucleotide frequencies at the three codon positions (CodonFreq = 2). The omega value was assumed to be the same across all codons in the sequence (model = 0). Omega and kappa were set to be estimated with the following initial values: fixed_kappa = 0, kappa = 1, fixed_omega = 0, omega = 1, Nsites=0. Estimated omega was considered reliable when dS had values between 0 and 1.2 as originally proposed by Koonin and co-workers [1]. PAML was run 10 times for each omega estimation and compared for consistency. We used values with the lowest log-likelihood score (lnL).Only those pairs of genomes for which more than 60% of genes in the reference genome had reliable omega values were taken for further analysis. This was to limit the distance between analyzed species and resulted in robust mutation rates for omega estimation. The preceding steps provided data for 8 out of 13 bacterial species with essentiality information in DEG (see Table 1).

**Estimation of evolutionary rate (omega based; dN/dS)**

We used genomes selected in the previous stage (those with at least 60% of genes in the reference genome having reliable omega) for omega calculations. Again we used data from ATGC where orthologs where clustered into groups with specific COG_id. We filtered out clusters of orthologs with less than three sequences. We used PAML model 0 (other setting were the same as above) to calculate omega for each cluster (MSA) of orthologs (CDS’s). We used PHYLIP package (NJ method with default parameters) to generate a single Neighbour-Joining tree for each MSA. We removed cases when dS was higher than 5, as this indicated an unreliable omega value. We also removed the cases with predicted occurrence of positive selection (omega > 1).

**Recombination analysis**

For each cluster of orthologs with reliable omega we checked whether it underwent recombination. We used two tools: Geneconv and Phi (with three different methods for recombination detection). As the recombination algorithms are known to be oversensitive (prone to false positives detection) we assigned the recombination event to a given cluster of orthologs if it was detected by Geneconv and any methods from the Phi package. In each test (Geneconv and three methods from the Phi package) we applied Benjamini-Yekutieli correction for multiple testing under dependency. Distribution of recombination events across eight analyzed bacteria is presented in Table 2.

**Assessment of dN/dS differences**

For each species, we assessed the statistical difference of relative speed of evolution between the three aforementioned sets of genes (all, essential and potential wide-spectrum drug targets). We used the Mann-Whitney U test. The p-values for a difference between sets were calculated using R implementation of the test (wilcox.test function; two sided). In our statistical approach we tested 24 hypotheses (three groups together for eight species). We corrected our p-values using the FDR approach (Benjamini-Yekutieli correction for multiple testing approach). Results are presented in Figure 1 and 2 [see also additional file 3 for statistical details].

**Omega issues**

Presented results complement the pN/pS analysis. As we pointed out in the discussion, there are certain conditions under which omega can be considered a reliable estimate of evolutionary rate.

In our approach we calculated omega for clusters of closely related species and strains. In fact omega was designed for interspecies analyzes. However, bacteria seem unique in this regard. Definition of bacterial species has been under discussion for the past twenty years. We are still not sure whether a given subspecies is not in fact another species [2,3]. *Francisella novicida* U112 strain, used in this study is an example of such ambiguity [4].Just a few years ago it was considered a subspecies of *Francisella tularensis* (*Francisella tularensis* subsp. *novicida* U112). Thus in our analysis instead of not-so-obvious interspecies data, we estimated (using whole-genome pairwise alignments of ortholougs sequences) evolutionary proximity and filtered out not divergent enough species/strains (dN = 0) and too divergent species/strains (dS> 1.2).

**References**

1. Novichkov PS, Ratnere I, Wolf YI, Koonin EV, Dubchak I: **ATGC: a database of orthologous genes from closely related prokaryotic genomes and a research platform for microevolution of prokaryotes**. *Nucleic Acids Res* 2009, **37**(Database issue):D448-454.

2. Georgiades K, Raoult D: **Defining pathogenic bacterial species in the genomic era**. *Front Microbiol*2010, **1**:151.

3. Staley JT: **The bacterial species dilemma and the genomic-phylogenetic species concept**. *Philos Trans R SocLond B BiolSci*2006, **361**(1475):1899-1909.

4. Splettstoesser WD, Seibold E, Zeman E, Trebesius K, Podbielski A: **Rapid differentiation of Francisella species and subspecies by fluorescent in situ hybridization targeting the 23S rRNA**. *BMC Microbiol*2010, **10**:72.

# Figures

**FIGURE LEGENDS**

## Figure 1 - Evolutionary rate differences

Evaluation of evolutionary rate differences between three sets of genes of interest: ALL -all genes, ESS - essential ones and APD - approved drug targets). Evolutionary rate was estimated using (dN/dS ratio). In this case dN/dS values were compared using Mann-Whitney U test (wilcox.test in R language, two sided hypothesis tested). Boxplots of means of dN/dS with 95% confidence intervals are presented (number of genes in given set are shown in brackets). Result for four species from *Enterobacteriaceae*. Abbreviations: ECO: *Escherichia coli*, STM – *Salmonella typhimurium*, PAU – *Pseudomonas aeruginosa*, FTN – *Francisella novicida*

#
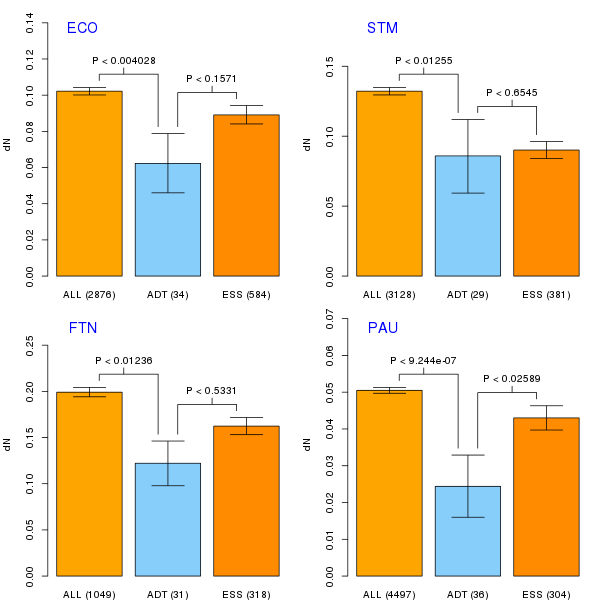


## Figure 2 - Evolutionary rate differences

Evaluation of evolutionary rate differences between three sets of genes of interest: ALL -all genes, ESS - essential ones and APD - approved drug targets). Evolutionary rate was estimated using (dN/dS ratio). In this case dN/dS values were compared using Mann-Whitney U test (wilcox.test in R language, two sided hypothesis tested). Boxplots of means of dN/dS with 95% confidence intervals are presented (number of genes in given set are shown in brackets). Result for four species not from *Enterobacteriaceae* taxon. Abbreviations: HPY: *Helicobacter pylori*, HIN – *Haemophilus influenzae*, SAO – *Staphylococcus aureus*, SPN – *Streptococcus pneumoniae*


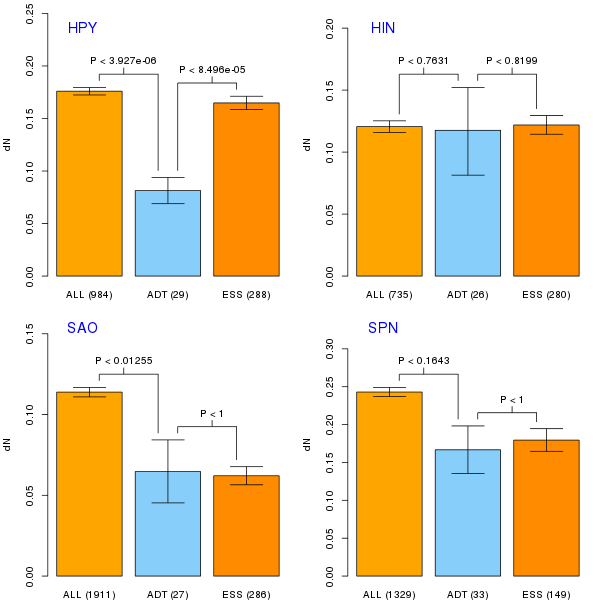


# Tables

## Table 1 - Summary of the data used at dN/dS analysis

| **Reference genome (strain with experimental data about essentiality; NCBI Taxonomy ID in brackets)** | **Others strains/species with complete pairwise alignments with reference genome (NCBI Taxonomy ID’s)** | **The number of all genes** | **The number of essential genes** | **The number of drug targets** |
| --- | --- | --- | --- | --- |
| *Escherichia coli K12 (*83333) | 155864, 198214, 198215, 199310, 209261, 220341, 290338, 290339, 295319, 300267, 300268, 300269, 316407, 321314, 331111, 331112, 362663, 364106, 373384, 386585, 399742, 405955, 99287 | 4294 | 771 | 41 |
| *Francisella novicida U112* (401614) | 119857, 177416, 393011, 393115, 418136, 458234 | 1719 | 391 | 34 |
| *Haemophilus influenzae Rd KW20* (71421) | 262727, 262728, 281310, 374927, 374928, 374930, 374931, 374932, 374933, 375063, 375177, 375432 | 1581 | 477 | 39 |
| *Helicobacter pylori 26695 (*85962) | 357544, 85963 | 1576 | 336 | 30 |
| *Pseudomonas aeruginosa UCBPP-PA14 (*208963) | 208963, 381754 | 5892 | 335 | 40 |
| *Salmonella typhimurium LT2 (*99287) | 155864, 198214, 198215, 199310, 209261, 220341, 290338, 290339, 295319, 300267, 300268, 300269, 316407, 321314, 331111, 331112, 362663, 364106, 373384, 386585, 399742, 405955, 83333 | 4425 | 235 | 41 |
| *Staphylococcus aureus N315 (*93061) | 158878, 158879, 196620, 273036, 282458, 282459, 359786, 359787, 367830, 418127, 426430, 93062 | 2892 | 351 | 35 |
| *Streptococcus pneumoniae TIGR4 (*170187) | 171101, 373153, 406556, 406557, 406558, 406559, 406560, 406561, 406562, 406563 | 1965 | 195 | 37 |

A summary of the data used in the dN/dS analysis.

## Table 2 - Distribution of recombination events in analyzed species

| **Reference genome (strain with experimental data about essentiality; NCBI Taxonomy ID in brackets)** | **The number of orthologous groups analyzed** | **The number of orthologous groups with recombination detected** |
| --- | --- | --- |
| *Escherichia coli K12 (*83333) | 3865 | 292 |
| *Francisella novicida U112* (401614) | 1464 | 7 |
| *Haemophilus influenzae Rd KW20* (71421) | 1524 | 499 |
| *Helicobacter pylori 26695 (*85962) | 1328 | 29 |
| *Pseudomonas aeruginosa UCBPP-PA14 (*208963) | 4880 | 29 |
| *Salmonella typhimurium LT2 (*99287) | 3919 | 227 |
| *Staphylococcus aureus N315 (*93061) | 2569 | 93 |
| *Streptococcus pneumoniae TIGR4 (*170187) | 1906 | 210 |
